# Use of Ecotoxicological Tools to Evaluate the Health of New Bedford Harbor Sediments: A Microbial Biomarker Approach

**DOI:** 10.1289/ehp.6934

**Published:** 2004-12-08

**Authors:** Timothy Ford, Jenny Jay, Anand Patel, Molly Kile, Phanida Prommasith, Tamara Galloway, Ross Sanger, Karen Smith, Mike Depledge

**Affiliations:** ^1^Department of Microbiology, Montana State University, Bozeman, Montana, USA; ^2^Department of Civil and Environmental Engineering, University of California Los Angeles, Los Angeles, California, USA; ^3^School of Public Health, Harvard University, Boston, Massachusetts, USA; ^4^Plymouth Environmental Research Center, University of Plymouth, Plymouth, United Kingdom

**Keywords:** metal-resistance genes, microbial diversity, RAMP, RFLP

## Abstract

We have been investigating microbial communities in sediments from New Bedford Harbor (NBH), Massachusetts, USA, for a number of years. NBH is a U.S. Environmental Protection Agency–designated Superfund site heavily contaminated with polychlorinated biphenyls, polycyclic aromatic hydrocarbons, and heavy metals. Microorganisms are thought to contribute to the fate and distribution of contaminants in NBH through a variety of mechanisms, including direct transformations and formation of soluble and insoluble species. Our more recent research has focused on changes in microbial community structure and function in response to exposure to toxic contaminants, with the ultimate goal of using microbes as ecotoxicological tools. Microbial diversity, as measured by restriction fragment-length polymorphism analysis, changes along pollution gradients, with an apparent increase in diversity at the most contaminated sites, concomitant with an increase in genetic relatedness. Current work on microbial communities examines the presence of arsenic-resistance genes in NBH isolates. In collaboration with the Plymouth Environmental Research Center, Plymouth University, United Kingdom, we have also used more conventional ecotoxicological approaches to examine the health of the NBH biota.

Participants of the “Roundtable Discussion on Biological Activity of Remediation Products” held at the Asilomar Conference on Bioremediation and Biodegradation, 9–12 June 2002, presented a framework for discussion that highlights the limitations of monitoring techniques for site remediation (Figure 1) (Ford et al. 2002). Research at the New Bedford Harbor (NBH), Massachusetts, USA, a U.S. Environmental Protection Agency (U.S. EPA)–designated Superfund site, has focused partly on developing ecological biomarkers of contaminant exposure for use in monitoring remediation of contaminated sites.

## The New Bedford Harbor Superfund Site

**Figure 2:**
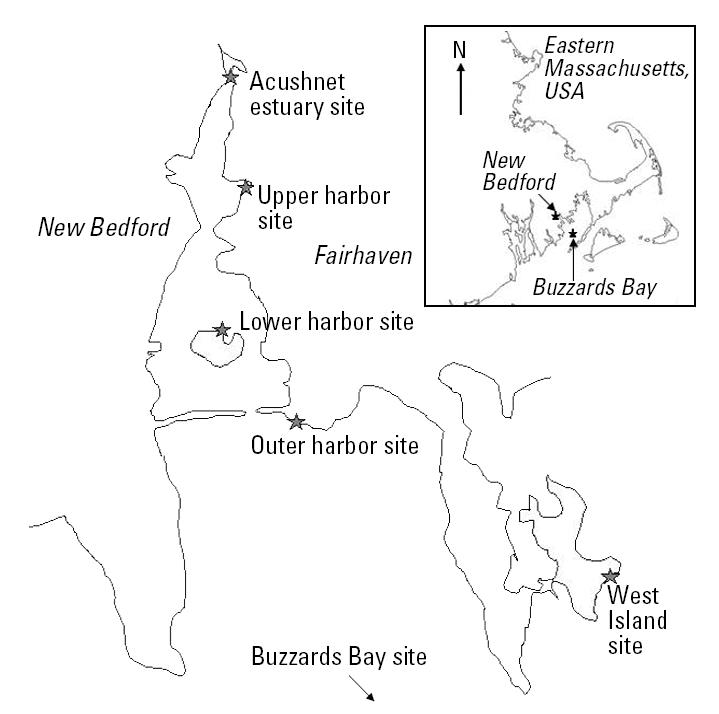
Map of New Bedford Harbor sites.

**Figure 3:**
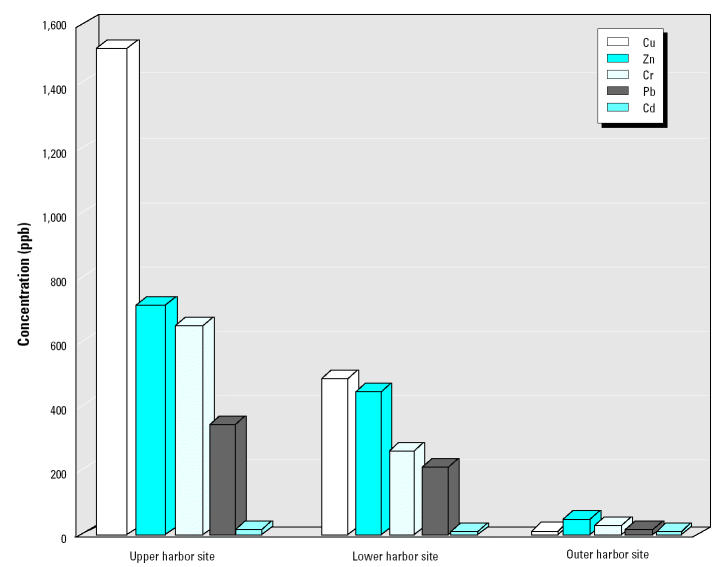
Metals in New Bedford Harbor. Figure adapted from Ford et al. (1998).

New Bedford Harbor is located approximately 40 miles south of Boston (Figure 2). The harbor is a poorly flushed estuary with a long history of metal contamination. Our early research focused on characterizing metal distribution throughout the harbor sediments (Ford et al. 1998; Shine et al. 1995). Sediment from the upper harbor has much higher concentrations of copper, zinc, chromium, lead, and cadmium compared with the lower and outer harbor (Figure 3) (Ford et al. 1998).

The harbor has also been heavily contaminated with polychlorinated biphenyls (PCBs) from capacitor manufacturers in the 1930s and 1940s. PCB usage peaked at about 2 million pounds/year between 1973 and 1975. Sediment concentrations of PCBs were measured as high as 100,000 ppm in the upper harbor. In 1964 a hurricane barrier was constructed that restricted the flushing rates of the estuary and altered flow patterns. Eighteen thousand acres of the harbor and outer bay are currently closed to commercial and recreational fishing (Nelson et al. 1996).

In 1987 a pilot study was conducted to examine dredging and disposal options to remove the most contaminated sediments from the harbor. In 1990 a Record of Decision was signed to remove approximately 10,000 cubic yards of sediment with PCB concentrations > 4,000 ppm. Dredging of this hot spot was completed in the fall of 1995. However, the dredged sediments currently remain in a confined disposal facility at the side of the harbor, prior to eventual removal for land-filling out of state.

Over the years, a number of ecological studies have examined the effects of contaminant exposure on NBH biota. For example, total PCBs in mummichogs have been measured by the U.S. EPA Narragansett Laboratories at 300 μg/g dry weight in the upper harbor, with decreasing concentrations toward Buzzards Bay (Nelson et al. 1996). Black et al. (1998) found concentrations of PCBs in mummichog livers of 35 μg total PCBs/g dry weight associated with increased mortality, reduced survival of progeny, and greater spinal abnormalities.

## Monitoring Remediation of Contaminated Sediment

This raises the question: How do we evaluate remediation of contaminated sediment? The U.S. EPA has mandated a long-term monitoring program for NBH and the surrounding waters that includes the collection and analysis of sediment chemistry, bioaccumulation tests, and sediment characterization (acute toxicity tests, grain size and texture, and benthic community structure) (Nelson et al. 1996). For example, using the 75% benthic community abundance measure, the upper harbor is dominated by the polycheate *Streblospio benedicti*, whereas the lower harbor is dominated by the clam *Mulinia lateralis*; the outer harbor is dominated by *Ostracoda* (Nelson et al. 1996). A long-term remediation strategy would therefore be evaluated based on a reduction in concentration of sediment contaminants and a return to community abundance that reflects a less-contaminated state. A number of problems occur, however. Measures of sediment chemistry do not reflect the bioavailable portion of contaminants present, and in fact, a decrease in contaminant concentration could be accompanied by an increase in bioavailability through an inappropriate remediation strategy (e.g., addition of nutrients to stimulate microbial activity; oxygenation of sediments through dredging). Community abundance and diversity is also problematic. For example, Nacci et al. (1999) have shown that *Fundulus heteroclitis* indigenous to this site actually have an inherited tolerance that allows them to survive in large numbers.

Our program focuses on using rapid ecotoxicological approaches to monitoring the health of NBH. This includes development of microbial biomarkers of contaminant exposure and the application of the rapid assessment of marine pollutants (RAMP) technique developed at the Plymouth Environmental Research Center in the United Kingdom.

The specific aims of our research program are to *a*) develop a suite of microbial molecular biomarkers that will indicate the bioavailability of contaminants and the potential for adverse ecological effects; *b*) evaluate the physiological and biochemical responses in the biota to validate the microbial biomarker approach and at the same time provide additional tools to characterize the stress of the aquatic ecosystem; and *c*) in the long term, provide multiple probes for the analysis of ecosystem health, which can be used as monitoring or screening tools for environmental decision makers who are evaluating remediation alternatives for contaminated aquatic sediments.

It should be noted that the information reported in this article reflects our progress to date and does not seek to provide answers for all the specific aims of our research program. Analysis of gene presence alone in highly contaminated marine sediments is extremely complex. The eventual goal of monitoring gene expression for a suite of microbial biomarkers requires a long-term and research-intensive program.

## Past and Current Research

### Microbial biomarkers.

**Figure 4:**
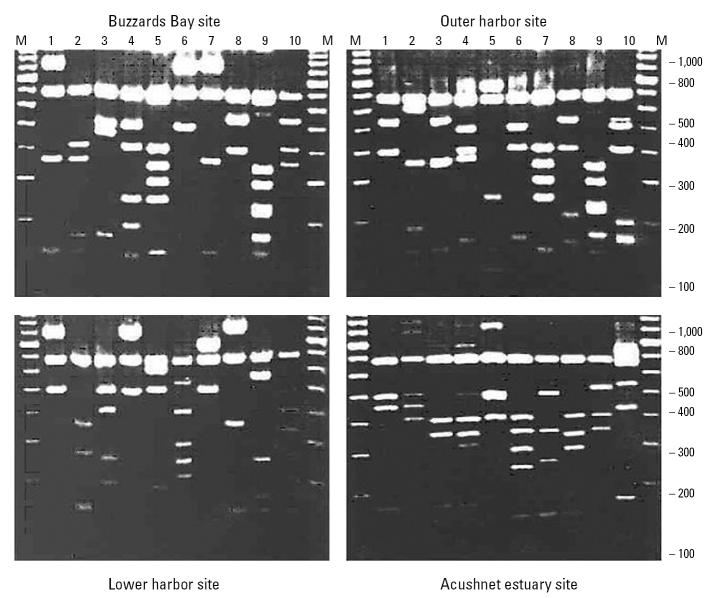
Examples of unique RFLP patterns from New Bedford Harbor area sediments. M designates the marker standard. The 684 base-pair fragment present in all lanes was generated as a result of *RSA*1 endonuclease digestion of the pCRII vector and used as an internal reference. Figure modified from Sorci et al. (1999).

Our initial approach to developing microbial biomarkers of exposure to bioavailable contaminants focused on examination of microbial molecular diversity along pollution gradients. If contaminants are not bioavailable, then diversity should not be affected by their presence. We evaluated species diversity by extracting DNA from sediment and subjecting it to restriction fragment length polymorphism (RFLP) analysis of the 16S rRNA genes (Figure 4). We then applied a cluster analysis to the resulting fragment patterns (known as operational taxonomic units) to compare genetic distances. Although our results suggested greater diversity at contaminated sites relative to less-contaminated sites, they also suggested an increased genetic relatedness. This may be consistent with a more constrained (stressed) environment with a wide diversity of organic carbon sources (Sorci et al. 1999).

As with higher organisms, a contaminated environment is likely to select for contaminant-resistant organisms, and measurements of diversity may be misleading. This certainly appears to be the case in NBH, where diversity increases with higher contaminant (and organic carbon) loads. An alternative approach that reflects ongoing research in our laboratory is to look for the presence of specific genes that convey resistance to toxic metals or the ability to degrade toxic organic compounds. For NBH, both approaches are possible. If these genes are present, this provides at least preliminary evidence that the organisms may be exposed to bioavailable contaminants. The eventual goal of the research program is to quantitatively assess both the presence of selected genes, on the assumption that copy number will increase with increasing pollution, and their expression (see “Research Directions”).

### Evaluation of metal resistance.

As a starting point for this research, we exploited the ability of bacteria to develop metal resistance as a microbial biomarker. The genes that convey arsenic (As) resistance were used as a model, as they have been well characterized in the literature. The arsenic resistance operon, known as *ars*, encodes a detoxification system that includes reduction of As(V) to As(III) by the soluble reductase ArsC, followed by extrusion from the cell by the membrane pump ArsB alone or in conjunction with ATPase ArsA (Rosen 1999; Silver 1998; Xu et al. 1998). The *ars* operon has been found on gram-negative and gram-positive bacteria (Diorio et al. 1995; Silver 1998) on plasmids (Kaur and Rosen 1992), transposons (Summers 1992), and the chromosome of *Escherichia coli* (Carlin et al. 1995) and *Thiobacillus ferrooxidans* (Butcher et al. 2000). *Ars* genes have been observed in *Desulfovibrio* Ben-RA, isolated from an Australian reed bed (Macy et al. 2000); *Pseudomonas fluorescens* MSP3, isolated from seawater (Prithivirajsingh et al. 2001); and aerobic bacteria from sewage and As-enriched creek waters (Saltikov and Olson 2002). However, the prevalence of these genes in aerobes and anaerobes from a range of environmental systems is unknown.

Shallow water sediment samples (18 μg/g As, dry weight) were collected from a contaminated NBH field site using a sterilized gravity coring device (Wildco, Inc., Buffalo, NY) and placed on ice for transfer to the laboratory. For aerobic microbiology, bacteria were extracted from sediment (Engelen et al. 1998), serially diluted, and plated onto marine agar plates (Difco Diagnostic Systems, Sparks, MD) amended with concentrations of As (as sodium arsenate) ranging from 0.1 to 10 mM. For anaerobic microbiology, a portion of the core was transferred to the anaerobic chamber (5% hydrogen, 15% carbon dioxide, 80% nitrogen). Triplicate aliquots were diluted in anaerobic solutions of 0.5 M sodium chloride and 0.05 M Tris buffer (pH 7.8) and plated onto basal salt sulfate-reducing media (with lactate, acetate, pyruvate, and butyrate as carbon sources) amended with As (as sodium arsenate) ranging from 0.1 to 10 mM. Individual colonies of As-resistant bacteria were picked (from plates containing arsenate concentrations that inhibited growth and diversity of bacteria) and transferred at least 3 times per isolate before analysis on plates containing 10 and 1 mM arsenate for aerobes and anaerobes, respectively.

Marine agar (for aerobic isolates) and sulfate-reducing agar (for anaerobic isolates) plates were poured with sodium arsenate concentrations of 0, 10, 20, 50, 100, and 150 mM. Aerobic and anaerobic isolates were allowed to grow on the plates at room temperature for 7 and 14 days, respectively. Tolerance was determined as the highest concentration in which growth occurred on both duplicate plates.

Isolates were genetically grouped using RFLP. Cells were lysed by heat, sonication, and alternate rapid freeze/thaw. The 16S rRNA gene was amplified by polymerase chain reaction (PCR) from the crude DNA, RFLP was performed using the restriction endonuclease known as *Hha*I, and RFLP profiles were grouped manually, allowing approximately 5% variation in fragment length within groups. Of 200 bacterial species isolated, RFLP grouping revealed 14 groups of aerobes and 9 groups of anaerobes.

Plasmid DNA was extracted using a modified phenol/chloroform extraction (25:42:1 phenol/chloroform/isoamyl alcohol) (Ausubel et al. 1995), which denatures and eliminates chromosomal DNA. Genomic DNA was also extracted by a phenol/chloroform method designed to extract total cellular DNA that would yield primarily chromosomal DNA. Although the plasmid extraction method affords virtual certainty that PCR-amplified genes from such samples are located on plasmids, the chromosomal DNA extraction method is less definitive. Although we expect plasmid DNA to be greatly reduced in these extracts, it is possible that large plasmids could be extracted along with the chromosome.

Nested primer sets were developed for the amplification of *arsA*, *arsB*, and *arsC*, the three genes on the *ars* operon that encode for ArsA ArsB, and ArsC, respectively (Table 1). *E. coli* with the pUM3 plasmid (kindly provided by B. Rosen) known to contain the *ars* operon was used as a positive control for plasmid extraction and PCR, and *E. coli* K12 was used as the chromosomal DNA positive control. Each PCR reaction contained 25 μL HotStarTaq Master Mix (Qiagen, Valencia, CA), 0.1 μM of each primer (Great American Gene Company, Ramona, CA), 0.2 μg of template DNA, and distilled water to give a final volume of 50 μL. Amplification of *arsA*, *arsB*, and *arsC* were multiplexed in the same reaction and run in a Geneamp 2400 thermocycler (PerkinElmer, Norwalk, CT) (15 min at 95°C, 30 cycles of 1 min at 94°C, 1 min at 50°C, 1 min at 72°C, 10 min extension at 72°C, and hold at 4°C).

**Figure 5:**
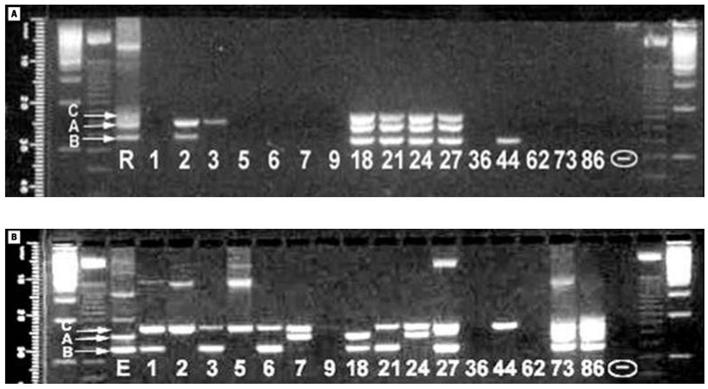
Electrophoresis gels showing PCR-amplified products of *arsA*, *arsB*, and *arsC* in (*A*) genomic and (*B*) plasmid DNA extracts of As-resistant New Bedford Harbor isolates. Numbers refer to specific isolates listed in Table 2.

For 16 aerobic isolates representing 10 RFLP groups, and 7 anaerobes representing 4 RFLP groups, plasmid and chromosomal DNA were screened for the presence of *arsA*, *arsB*, and *arsC* (Figure 5; Table 2). *ars* Genes were observed in chromosomal extracts of all but three strains tested and 10 plasmid DNA extracts. As previously described, the chromosomal DNA preparation is likely to contain plasmid DNA. However, in six cases, *ars* genes were detected in plasmid DNA preparations and not in chromosomal DNA preparations, strongly suggesting that they are present on plasmid DNA. Conversely, in 15 cases, *ars* genes were detected in chromosomal DNA extracts but not in plasmid DNA extracts, suggesting that the genes are either present on chromosomal DNA or on very large plasmids not extracted in the plasmid DNA methodology. This distinction is important both from an ecological viewpoint and for the use of genetic markers as indicators of contaminant stress. Mobile genetic elements may be more rapidly disseminated within the sediment microbial communities in response to bioavailable contaminants and hence may provide a better indicator of contaminant exposure than chromosomal DNA (Ford 2000). Further hybridization studies are clearly warranted on these isolates to better distinguish between chromosomal and plasmid genes.

In 11 cases, all three *ars* genes were observed together; however, in a number of cases only one or two of the genes were observed. Because the genes are part of the same operon and are regulated together, the absence of observed *arsB* or *arsC* along with other *ars* genes is probably indicative of variations in the gene that decreased the homology with our primer set. Because the arsenite extrusion pump (ArsB) can function alone, absence of observable *arsA* could indicate nonhomology, or an operon without *arsA*. In 20 of the 22 cases when any of the *ars* genes were observed, *arsC* was present.

Arsenic tolerance levels were determined for each isolate (Table 2). Tolerance range is from 20 to 150 mM, There is no clear relationship between tolerance level and the prevalence of the *ars* genes. All the anaerobes were able to tolerate at least 50 mM As added to the medium. Tolerance is likely dependent on the speciation in the medium, which we did not measure.

This work shows that the *ars* genes are prevalent in NBH sediments among both aerobic and anaerobic bacteria with a diverse group of 16S rRNA RFLP patterns. This has important implications for As cycling, as the form of As extruded by this detoxification mechanism [As(III)] is the more mobile and toxic form. The nested primer method described here is capable of amplifying these genes from a contaminated site. These findings may be applied to the use of *ars* genes as a biomarker for bioavailable As in the field.

As mentioned previously, NBH is contaminated with a wide range of metals and organics, and there are many potential gene targets to use as microbial biomarkers. The As-resistance system is only one example, and our laboratory is currently investigating the presence of a number of other genes (see final section). However, presence alone is likely to be a poor indicator of exposure to bioavailable contaminants, and our current focus is on optimizing RNA extraction from NBH sediments for evaluation of gene expression.

### Rapid assessment of marine pollution.

**Figure 6:**
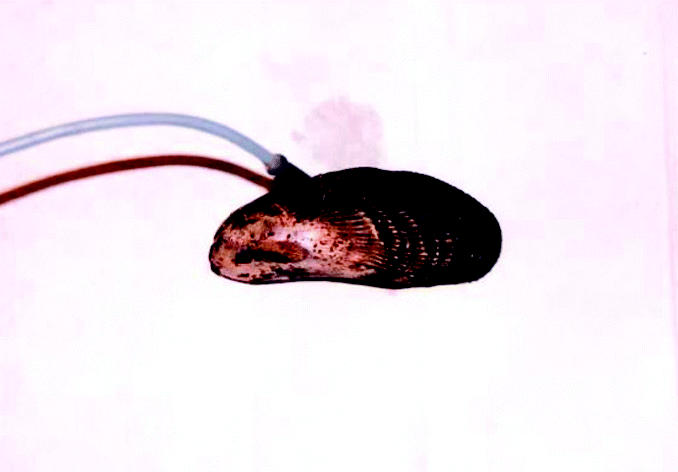
Atlantic ribbed mussels were used as the bioindicator organism for New Bedford Harbor. Their heart rate was monitored using an infrared heart rate monitor attached to the surface of the mussel’s shell. Photograph courtesy of Ross Sanger, University of Plymouth.

The second component of our research program is to examine more traditional ecotoxicological indicators in higher organisms using the RAMP approach (Wells et al. 2001). The eventual aim is to correlate responses in higher organisms with the microbial approach. For example, if a genotoxic response to a specific pollutant (or mixture) in an invertebrate species increases along a pollution gradient, we might expect increased expression of a specific gene in the microbial population. In these studies we have focused on the biochemical and physiological activity of the Atlantic ribbed mussel, *Geukensia demissa*, which is extremely common in NBH and the surrounding coastal areas of Buzzards Bay. *Geukensia* lives partially within the surficial sediments, making it an excellent candidate for this study, as it is directly exposed to high levels of sediment contamination (Figure 6).

The RAMP approach was developed at the Plymouth Environmental Research Center in the United Kingdom. This approach combines chemical residue analysis with measurement of a range of biological responses to determine the ecological health of a marine ecosystem. Approaches include *a*) evaluating the physiological status of the organism by monitoring its heart rate or condition index; *b*) evaluating genotoxicity by observing micronucleus formation; *c*) evaluating cellular status by measures of cell viability and lysosomal integrity; and *d*) evaluating immunotoxicity through measures of spontaneous cytotoxicity.

**Figure 7:**
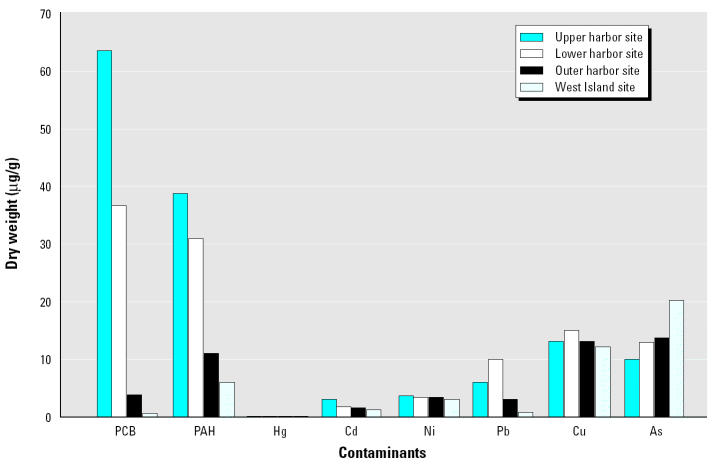
Mussel tissue burdens of PCBs, PAHs, and selected metals (Hg, Cd, Ni, Pb, Cu, As). Figure adapted from Galloway et al. (2002).

**Figure 8:**
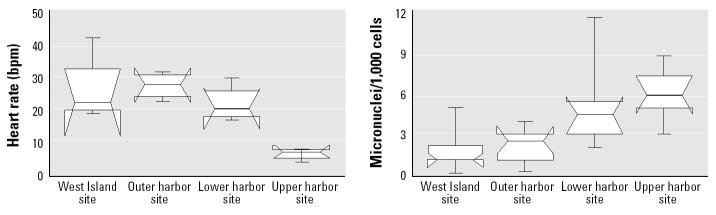
Examples of RAMP assays applied to Atlantic ribbed mussels from New Bedford Harbor. Figure adapted from Galloway et al. (2002).

A summary of our findings from the RAMP survey found that PCBs and polycyclic aromatic hydrocarbons (PAHs) in the mussel tissue were greatest at the inner harbor site and decreased along a pollution gradient out toward the control site in Buzzards Bay (Figure 7). Chromosomal damage was greatest at the most highly polluted sites, and immune function, heart rate, and cell viability all decreased with increasing pollution (Galloway et al. 2002). Figure 8, adapted from Galloway et al. (2002), illustrates this relationship for heart rate and chromosomal damage. Significant differences in PCB and PAH tissue residues were detected among sites using immunoassay techniques (RaPID assay; Ohmicron Environmental Diagnostics, Inc., Newtown, PA). However, no significant differences were observed in metal concentrations in mussel tissues (copper, cadmium, lead, As, mercury, and nickel) throughout the area. Multivariate canonical correlation analysis indicated that PCB and PAH concentrations were strongly associated with biomarkers of genotoxicity (micronucleus formation), immunotoxicity (spontaneous cytotoxicity), and physiological impairment (heart rate) (Galloway et al. 2002).

## Research Directions

Our current goal is to target other resistance or catabolic genes that may be more prevalent than *ars* genes to use as microbial biomarkers. We have begun to evaluate sediments for the presence of biphenyl-degrading genes; the biphenyl degrading (*bph*) gene cluster implicated in the degradation of PCBs to chlorobenzoates through the 2,3-deoxygenation pathway (Furukawa and Kimura 1995). Increased copy number/expression of the *bph* genes is expected at the sediment–water interface as a response to both an overall increase in PCBs and an increase in the more readily biodegradable fraction (Erb and Wagner-Döbler, 1993). Once we have optimized our methodologies for detecting *bph* genes, we propose to expand the research in the following directions:

- Real-time PCR to detect changes in copy number of genes across a pollution gradient
- Extraction of mRNA from sediments to assess gene expression
- Develop fluorescent *in situ* hybridization (FISH) probes for mRNA to detect genes *in situ*
- As a long-term goal, be able to examine a number of metal-resistance systems and catabolic genes for PCB degradation concurrently, using DNA array technologies
- Validate all methodologies with more traditional biomarkers of exposure (RAMP).

### Microbial biomarkers as ecotoxicological tools.

Our long-term goal is to develop multiple probes to evaluate ecological health in marine ecosystems. Our approach will be to develop multiple FISH probes (or micro-arrays) to rapidly hybridize genes that are actively expressed in response to contaminant stress. These biomarkers should correlate with stress (biomarker) responses in higher organisms. We expect microbial biomarkers to be a rapid and sensitive measure of exposure to bioavailable contaminants, as microbes are ubiquitous in the environment, have no migratory behavior, and integrate responses to multiple stressors.

## Figures and Tables

**Figure 1:**
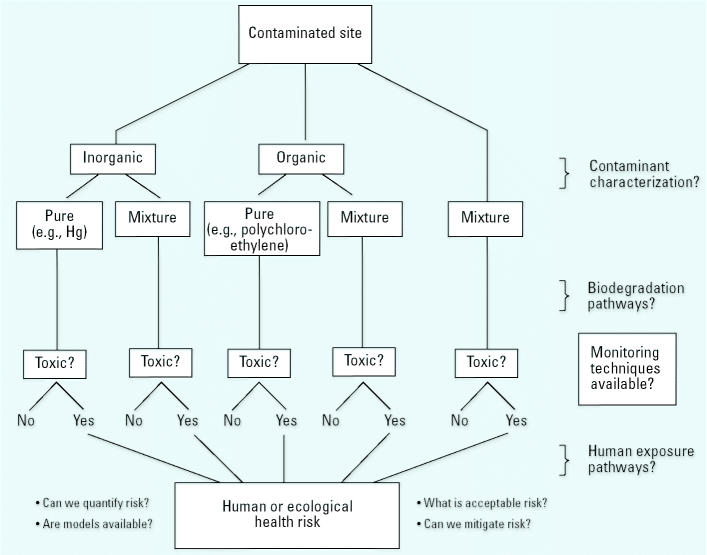
General model of the toxic risks of the remediation products of contaminated sites.

**Table 1:** Nested primer sets for *ars* genes.*^a^*

*ars* Gene	Outer primer sequence	Amplicon size	Inner primer sequence	Amplicon size
*arsA*	F*^b^* TAT TTC CTG CGC CAC GGC GAT	389	F CTG CTG GTC AGT ACC GAT	300
	R*^c^* GAA GGC GAA TGG TGT GAC		R GAT ATG GTC AAA CGT CAG	
*arsB*	F CCG GTG GTG TGG AAT ATT GT	409	F GTT GCT GGA TGA GTC AGG CT	259
	R ACT CCG TGA ATC CCA GTT		R GTA TCG GAA ATA CCG GC	
*arsC*	F CTG ATA TGA GCA ACA TCA CTA TTT	446	F ATC ATA ACC CAG CCT GC	341
	R ATT TCA GCC GTT TTC CTG CTT CA		R CTG CGC ATC CTG TAG GAT ARC	

a Primer set design was based on *ars* operon nucleotide sequence of resistance factor R773 (Chen et al. 1986). Primer3 software was used for primer design (version 1.0; Whitehead Institute for Biomedical Research, MIT, Boston, MA; Rozen and Skaletsky 2000).

b Forward.

c Reverse.

**Table 2:** Presence of *arsA*, *arsB,* and *arsC* on chromosomes and plasmids from 23 randomly selected aerobic and anaerobic New Bedford Harbor isolates and their respective As tolerance.

Isolate	Genomic			Plasmid			Tolerance (mM As)
	*arsA*	*arsB*	*arsC*	*arsA*	*arsB*	*arsC*	
A1		X	X				20
A2			X	X	X		20
A3		X	X		X		20
A5			X				20
A6		X	X				20
A7	X		X				20
A9			X				20
A18	X	X		X	X	X	100
A21	X		X	X	X	X	50
A24		X	X	X	X	X	150
A27	X		X	X	X	X	50
A36							–
A44			X	X			100
A62							50
A73	X	X	X				50
A86	X	X	X				50
N10							50
N11	X	X	X		X		50
N16	X	X	X			X	50
N1	X	X	X	X	X	X	50
N6	X		X		ND		150
N4*^a^*	X	X	X		X	X	ND
N3*^a^*	X	X	X			X	150

Abbreviations: A, aerobic isolate; N, anaerobic isolate; ND, not determined.

a Originally isolated on cadmium but also show arsenate resistance.
